# Association between body mass index and pain outcomes in elderly patients with chronic pain: A retrospective cohort study

**DOI:** 10.1007/s00540-025-03546-2

**Published:** 2025-07-15

**Authors:** Tamaki Aihara, Yusuke Nagamine, Masaki Kitahara, Takahisa Goto

**Affiliations:** 1 Department of Multidisciplinary Pain Management Clinic, Yokohama City University Medical Center, Yokohama, Japan; 2 Shinkawabashi Hospital, 1-15 Shinkawadori, Kawasaki-ku, Kawasaki-city, Kanagawa Japan; 3 Department of Anesthesiology and Critical Care Medicine, Yokohama City University Hospital, Yokohama, Japan

**Keywords:** Chronic pain, Body mass index, Elderly patient, Pain catastrophizing scale, Pain disability assessment scale

## Abstract

**Purpose:**

The purpose of this study was to investigate the association between body mass index (BMI) and changes in pain scores among elderly patients with chronic pain. The pain disability assessment scale (PDAS) and the pain catastrophizing scale (PCS) were employed as assessment tools.

**Methods:**

A single-center, retrospective cohort study was conducted at a university hospital multidisciplinary pain center from 2017 to 2020, involving 180 patients aged ≥ 65 years with noncancer pain persisting for at least 3 months. Patients were classified into three groups according to BMI: low (BMI < 18.5), standard (18.5 ≤ BMI < 25), and high (BMI ≥ 25). Initial, 3-month, and 6-month PDAS and PCS scores were collected and analyzed using mixed-effects models.

**Results:**

No significant differences were observed in PDAS scores across BMI groups. However, PCS scores were significantly higher in the low BMI group. Furthermore, no significant differences were detected in PDAS and PCS scores based on the interaction between BMI group and time point (month).

**Conclusion:**

Among elderly patients with chronic pain, the low BMI group exhibited a significantly higher PCS score, while PDAS scores did not vary based on the BMI group. No differences were detected in treatment-related changes over time across BMI groups.

**Supplementary Information:**

The online version contains supplementary material available at 10.1007/s00540-025-03546-2.

## Introduction

Chronic pain is a prevalent condition frequently encountered in elderly patients. One report indicated that 42.7% of the elderly population in Japan experiences some form of chronic pain [1]. The persistence of chronic pain in the elderly represents a critical issue that adversely affects quality of life and necessitates prolonged treatment, thereby consuming substantial medical resources.

Numerous studies have suggested an association between body weight and chronic pain. Research has identified obesity as a risk factor for various conditions, including low back pain, sciatica, and fibromyalgia [2–5]. In the context of post-herpetic neuralgia [6] and chronic widespread pain [7], both obesity and underweight have been documented as risk factors. A study examining the prevalence of chronic pain among Japanese men aged ≥ 50 years demonstrated a U-shaped relationship between chronic pain prevalence and BMI [8]. Additionally, a cross-sectional study involving elderly subjects reported that both obesity and underweight constitute risk factors for chronic pain [9].

Conversely, several studies investigating the efficacy of treatment for patients with fibromyalgia and chronic low back pain have reported no significant difference in treatment responses among normal weight, overweight, and obese patients [10, 11]. Some studies found less improvement in severely obese groups [12]. In the elderly, underweight status may pose a greater risk than obesity in terms of mortality and frailty [13]. Very few studies have quantitatively evaluated the effects of treatment on underweight patients. Consequently, a consensus on the relationship between treatment effects of chronic pain and BMI remains elusive.

In recent years, various scales have been employed in multidisciplinary pain management to quantitatively assess pain severity and treatment effectiveness. The pain disability assessment scale (PDAS) was developed in 1997 to evaluate physical movement and mobility in patients experiencing chronic pain [14, 15]. The Pain Catastrophizing Scale (PCS), developed by Sullivan et al. in 1995, is known to exhibit a strong correlation with pain severity [16]. This study aims to elucidate the relationship between BMI and changes in pain scores associated with multidisciplinary treatment among elderly patients with chronic pain, specifically utilizing the PDAS and PCS to evaluate physical and psychological aspects of pain, respectively, in this vulnerable population.

## Methods

### Design, setting, and patients

This was a single-center, retrospective cohort study conducted at a university hospital pain clinic, a multidisciplinary pain treatment center that primarily addresses chronic pain. Treatment plans are developed individually for each patient within the outpatient clinic setting. Patients are offered a combination of one or more of the treatment modalities, including the following: exercise therapy (providing basic exercise instructions by nurses and more individualized exercise therapy by physical therapists and occupational therapists, psychotherapy (cognitive-behavioral therapy by clinical psychologists, counseling, progressive muscle relaxation, etc.), pharmacological interventions, social interventions (e.g., new applications for long-term care insurance), nutritional guidance, acupuncture and moxibustion (administered by a dedicated acupuncturist), intramuscular stimulation, nerve blocks, and concurrent consultations with other departments.

Following were the inclusion criteria: (i) patients aged ≥ 65 years; (ii) those who attended the pain clinic between April 2017 and March 2020; (iii) those who presented with noncancer pain that persisted for at least 3 months; and (iv) those capable of participating in at least two medical interviews. Exclusion criteria encompassed patients with pain localized to the head and neck, cancer pain, acute postoperative pain, and those with incomplete interview data (lacking initial visit data or data from the third or sixth month).

### Exposure

BMI at the initial visit was calculated using height and weight measurements recorded by the nursing staff. BMI was categorized according to World Health Organization (WHO) standards[17]: low BMI group (BMI < 18.5), standard BMI group (18.5 ≤ BMI < 25), and high BMI group (BMI ≥ 25).

### Outcome

The primary outcomes comprised PDAS and PCS scores and their trends over time. PDAS and PCS scores were collected using medical interviews.

The PDAS is a self-administered questionnaire developed in Japan, consisting of 20 questions addressing daily physical movement and mobility on a 4-point scale. The questions include activities such as (Since an official English version of PDAS is not available, the following items have been translated by the authors for reference.) “climbing stairs,” “washing hair,” and “bending to pick up objects from the floor.” Each item is scored as follows: “No difficulty at all”: 0 points, “Slight difficulty”: 1 point, “Considerable difficulty”: 2 points, “Unable to perform due to severe pain”: 3 points. This method quantifies the degree of disability in daily activities, with higher scores indicating greater impairment. The PDAS yields a maximum score of 60 and a minimum of 0, with a cutoff value of 10 points. It does not assess pain localized to the head. Previous research has established that the minimal important difference (MID), corresponding to -2 on the numerical rating scale (NRS) for low back pain, is 6.71.

The PCS is also a self-administered questionnaire that measures the extent of pain-induced catastrophic thinking, comprising three subscales: rumination, magnification, and helplessness. The PCS consists of 13 items, such as “I worry all the time about whether the pain will end” and “I feel I can’t go on,” rated on a 5-point scale from “Not at all” (0 points) to “At all times” (4 points). Higher scores indicate increased catastrophic thinking. The PCS yields a maximum score of 52 and a minimum of 0, with a cutoff value of 30 points. Prior studies have indicated that the MID is 6.48, corresponding to -2 on the NRS for low back pain.

PDAS and PCS scores were collected during outpatient visits using a tablet computer, where patients entered their responses, and the scores were automatically calculated. For patients with visual impairments or those who had difficulty operating the tablet computer due to age, family members or administrative staff assisted in entering the responses.

### Covariates

Potential confounding variables included age, sex, charlson comorbidity index (CCI) [18], and mini mental state examination (MMSE) [19, 20]. As potential confounding factors that influence both exposure (BMI) and outcomes (PDAS and PCS), age, sex, physical comorbidities, and cognitive function were included. The CCI was used to evaluate physical comorbidities, and MMSE was used to evaluate cognitive function.

### Statistical analysis

The results were expressed as median (interquartile range [IQR]) or as numbers (%). A linear mixed-effects model was performed with individual patients as random intercepts. Fixed effects included the BMI category, the time point (month), and the interaction between the BMI category and time point (month), age, sex, CCI, and MMSE. Missing values were not imputed. Statistical analyses were performed using STATA version 17.0 (StataCorp, Texas, USA), with statistical significance set at a p-value of < 0.05.

### Ethical considerations

This study was approved by the ethics committee of the Yokohama City University Medical Center (approval number: B210500052, approval date: June 04, 2021, chairman: Shin Maeda). The requirement for informed consent was waived by the ethics committee. The opportunity to withdraw consent was communicated through a notice on the institution's website. All methods adhered to the principles outlined in the Declaration of Helsinki.

## Results

**Fig. 1:**
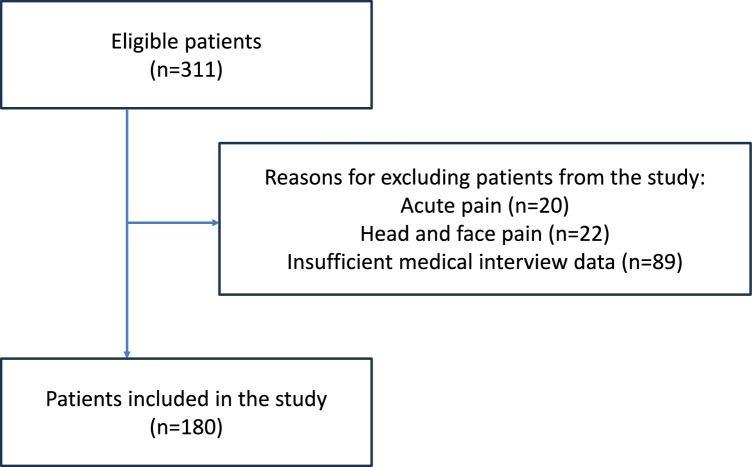
Study flow chart

Among 311 patients aged 65 years or older who visited the outpatient clinic from 2017 to 2020, 20 patients with acute pain, 22 patients with pain confined to the head and neck, and 89 patients with insufficient interview data were excluded, resulting in a final analysis of 180 patients (Fig. 1).

The characteristics of subjects in each group are detailed in Table 1. Among the 180 patients, 108 (60%) were female, with a median age of 74.7 years and a median BMI of 22.9. The distribution of patients and median BMI across BMI groups was as follows: 16 (8.9%) in the low BMI group (median BMI: 17.0), 114 (63.3%) in the standard BMI group (median BMI: 22.1), and 50 (27.8%) in the high BMI group (median BMI: 27.1).

**Table 1:** Baseline characteristics of the study population

	All		BMI < 18.5		BMI ≥ 18.5 and < 25		BMI ≥ 25
Number of patients	180		16(8.9)		114(63.3)		50(27.8)
Age (in years)	74.7[69.5–78.6]		76.3[74.2–78.8]		75.5[68.5–79.2]		71.3[68.5–75.1]
Male sex (%)	72 (40.0)		3(18.8)		44(38.6)		25(50.0)
BMI (kg/m^2^)	22.9[20.9–25.4]		17.0[10.1–18.1]		22.1[20.9–23.5]		27.1[26.1–28.4]
Diagnosis							
Myofascial pain	51(28.3)		1(6.3)		37 (32.5)		13 (26)
Osteoarthritis	32(17.8)		0(0)		15 (13.2)		17 (34)
Disuse syndrome	16(8.9)		5(31.3)		8 (7)		3 (6)
Chronic postoperative pain	16(8.9)		1(6.3)		9 (7.9)		6 (12)
Postherpetic neuralgia	15(8.3)		3(18.8)		10 (8.8)		2 (4)
Peripheral neuropathy	10(5.6)		1(6.3)		7 (6.1)		2 (4)
Cervical spondylosis	5(2.8)		0(0)		5 (4.4)		0 (0)
Vertebral compression fracture	5(2.8)		1(6.3)		3 (2.6)		1 (2)
Unclassifiable arthralgia	5(2.8)		0(0)		3 (2.6)		2 (4)
Unclassifiable low back pain	3(1.7)		0(0)		2 (1.8)		1 (2)
Lumbar spondylolisthesis or Spinal canal stenosis	2(1.1)		0(0)		2 (1.8)		0 (0)
Periarthritis of the shoulder	1(0.6)		0(0)		1 (0.9)		0 (0)
Other	19(10.6)		4(25.0)		12 (10.5)		3 (6)
Pain site							
Cervical region, shoulder, and upper limbs	19(10.6)		2 (12.5)		14 (12.3)		3 (6)
Thoracoabdominal region	14(7.8)		1 (6.3)		13 (11.4)		0 (0)
Back, Lumbar	26(52)		3 (18.8)		14 (12.3)		9 (18)
Gluteal regions, Lower limbs	111(61.7)		9 (56.3)		66 (57.9)		36 (72)
Whole body	10(5.6)		1 (6.3)		7 (6.1)		2 (4)
CCI scores	1[0–2]		1[0–2]		1[0–2]		1[0–2]
MMSE scores	28[26–29]		27[25–29]		28[26–29]		28.5[27–29]
History of alcohol consumption							
None	91(50.6)		10(62.5)		61(53.5)		20(40.0)
Past drinker	19(10.6)		3(18.8)		13(11.4)		3(6.0)
≤ 2 per week	34(18.9)		1(6.3)		19(16.7)		14(28.0)
≥ 3 per week	36(20.0)	2(12.5)		21(18.4)		13(26.0)	
History of smoking							
None	124(68.9)	13(81.3)		80(70.2)		31(62.0)	
Past smoker	49(27.2)	1(6.3)		31(27.2)		17(34.0)	
Current smoker	7(3.9)	2(12.5)		3(2.6)		2(4.0)	
Japanese long-term care insurance care-need level							
None	144(80.0)	12(75.0)		90(79.0)		42(84.0)	
Support level 1	9(5.0)	0(0.0)		6(5.3)		3(6.0)	
Support level 2	11(6.1)	2(12.5)		6(5.3)		3(6.0)	
Care-need level 1	8(4.4)	1(6.3)		5(4.4)		2(4.0)	
Care-need level 2	6(3.3)	1(6.3)		5(4.4)		0(0.0)	
Care-need level 3	2(1.1)	0(0.0)		2(1.8)		0(0.0)	
Care-need level 4, 5	0(0.0)	0(0.0)		0(0.0)		0(0.0)	
PDAS							
Score at initial visit	26[18–35]	32[25–40.5]		25.5[15–36]		25[20–30]	
Score at 3 months	21[13–31]	30[17–40]		20[11–31]		22.5[17–28]	
Score at 6 months	22[13–30]	22.5[20–30]		21[12–29]		25[14–30]	
PCS							
Score at initial visit	34.5[27.5–41.5]	42[37.5–46]		34[27–41]		34[27–40]	
Score at 3 months	32[23–39]	34[31–38]		31[23–39]		33[21–40]	
Score at 6 months	31[22–40]	34[32–37.5]		31[23–37]		31[21–41]	
Intervention							
Pharmacotherapy	144(80.0)	15(93.8)		85(74.6)		44(88.0)	
Exercise remedy	137(76.1)	11(68.8)		88(71.2)		38(76.0)	
Psychotherapy	9(5.0)	1(6.3)		6(5.3)		2(4.0)	
Consultation with other departments	52(28.9)	6(37.5)		32(28.1)		14(28.0)	
Acupuncture or intramuscular stimulation	27(15.0)	3(18.8)		17(14.9)		7(14.0)	
Nerve block	3(1.7)	0(0.0)		2(1.8)		1(2.0)	
Nutrition counseling	11(6.1)	1(6.3)		3(2.6)		7(14.0)	

Values are presented as median [interquartile range] or as numbers (%)

*BMI* body mass index, *CCI* charlson comorbidity index, *MMSE* mini mental state examination, *PDAS* pain disability assessment scale, *PCS* pain catastrophizing scale

Japanese long-term care insurance (LTCI) care need level is determined by the nursing care needs certification board. There are seven levels of LTCI need certificates according to the total daily estimated care minutes (support levels 1–2, care need levels 1–5) [35, 36]

**Fig. 2:**
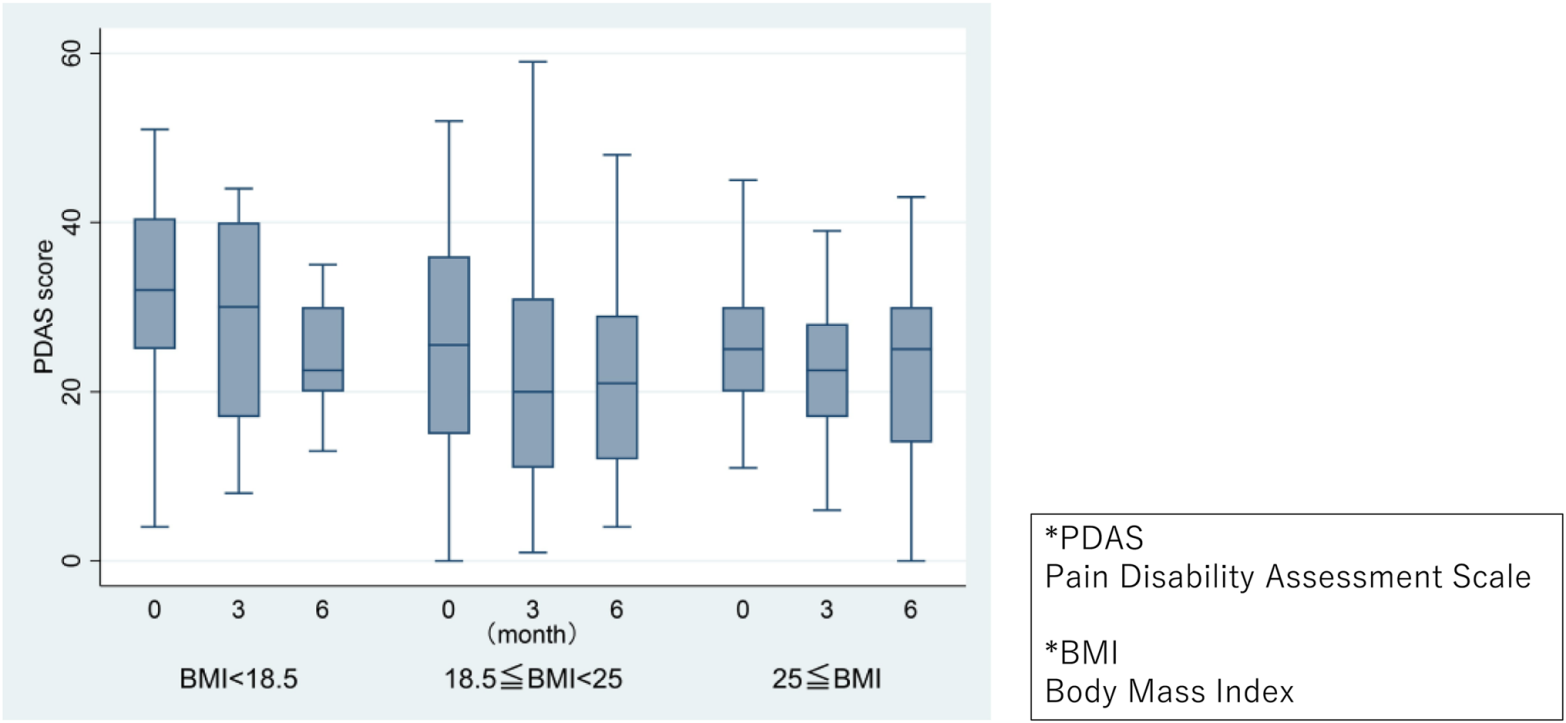
Change in PDAS scores over time per BMI group. *PDAS* pain disability assessment scale, *BMI* body mass index

Changes in PDAS scores over time by BMI group are illustrated in Fig. 2.

Analysis via mixed-effects modeling revealed significant reductions in PDAS scores at 3 and 6 months relative to the initial visit. No significant differences were observed in PDAS scores between BMI groups, and no significant differences were noted between BMI category and time point (month). Age was associated with significantly higher PDAS scores, whereas being male correlated with significantly lower scores; additionally, MMSE scores were associated with significantly higher PDAS scores (Table 2).

**Table 2:** Mixed effects model for PDAS

	Coefficient	*P* value	95% confidence intervals	
Time point (Month)				
3 months	−3.40	< 0.001	−5.04	−1.77
6 months	−2.70	0.006	−4.64	−0.76
BMI				
< 18.5	3.81	0.203	−2.06	9.69
≥ 25	2.33	0.267	−1.78	6.44
Confounding factor				
Age	0.37	0.009	0.09	0.65
Male sex	−4.66	0.005	−7.94	−1.38
CCI score	0.10	0.827	−0.77	0.97
MMSE score	−1.49	< 0.001	−2.22	−0.75
Interaction: “month × BMI group”				
“3 months” × “BMI < 18.5”	1.17	0.612	−3.37	5.71
“3 months” × “BMI ≥ 25”	1.61	0.314	−1.52	4.74
“6 months” × “BMI < 18.5”	2.10	0.463	−3.51	7.72
“6 months” × “BMI ≥ 25”	2.54	0.150	−0.92	6.01

*BMI* body mass index, *CCI* charlson comorbidity index, *MMSE* mini mental state examination, *PDAS* pain disability assessment scale

**Fig. 3:**
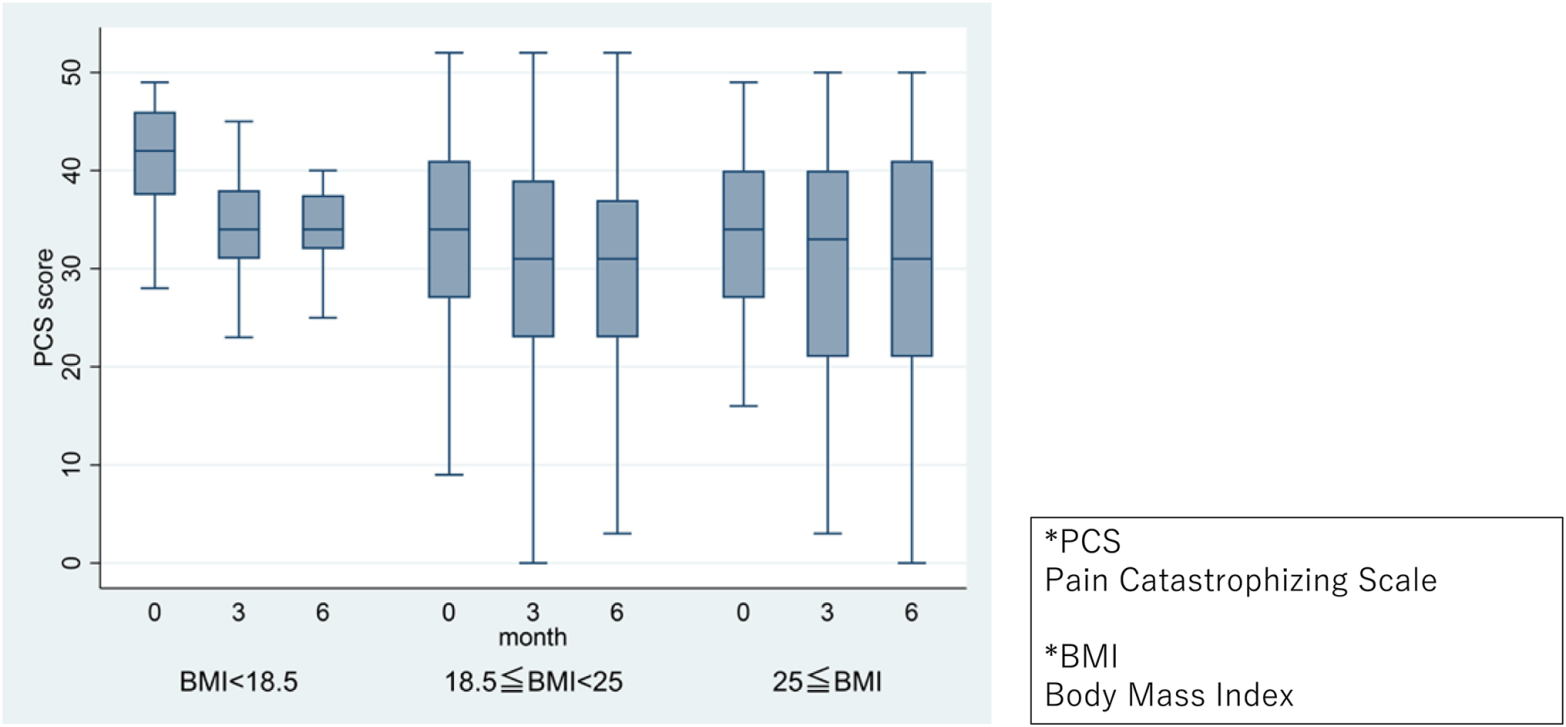
Change in PCS scores over time per BMI group. *PCS* Pain catastrophizing scale, *BMI* body mass index

Variations in PCS scores across time by BMI group are depicted in Fig. 3. Mixed-effects model analysis indicated significant reductions in PCS scores at 3 and 6 months compared to the initial visit, with the low BMI group displaying significantly higher PCS scores. No significant differences were identified in PCS scores regarding the interaction between BMI category and time point (month). Across all variables, MMSE scores were significantly associated with increased PCS scores (Table 3).

**Table 3:** Mixed effects model for PCS

	Coefficient	*P* value	95% confidence intervals	
Time point (Month)				
3 months	−3.79	< 0.001	−5.52	−2.06
6 months	−3.78	< 0.001	−5.82	−1.74
BMI				
< 18.5	6.59	0.022	0.95	12.22
≥ 25	0.53	0.794	−3.41	4.47
Confounding factor				
Age	−0.02	0.908	−0.28	0.25
Male sex	−0.32	0.839	−3.40	2.76
CCI score	−0.36	0.380	−1.18	0.45
MMSE score	−0.91	0.010	−1.60	−0.22
Interaction: “month × BMI group”				
“3 months” × “BMI < 18.5”	−3.05	0.210	−7.83	1.72
“3 months” × “BMI ≥ 25”	1.34	0.426	−1.96	4.64
“6 months” × “BMI < 18.5”	−2.33	0.439	−8.22	3.57
“6 months” × “BMI ≥ 25”	1.98	0.287	−1.66	5.62

*BMI* body mass index, *CCI* charlson comorbidity index, *MMSE* mini mental state examination, *PCS* pain catastrophizing scale

Subgroup analyses were performed for treatment interventions, including exercise remedy and pharmacotherapy. However, due to the small number of participants, subgroup analyses were not performed for acupuncture and psychotherapy. The results are presented in Supplementary Tables 1–4. The overall trend for PDAS remained largely unchanged. For PCS, the association with BMI was not statistically significant in the pharmacotherapy ( +) subgroup. In the exercise remedy ( +) subgroup, low BMI showed a statistically significant association with PCS scores.

The results from the mixed-effects model analyses for each PCS subscale are presented in Supplementary Tables 5–7. The helplessness subscale manifested significantly higher scores in the low BMI group, while no significant differences were identified among other PCS subscales across BMI groups. Among PCS subscales, only the catastrophizing component showed a significant decrease in the interaction of “3 months” × “BMI < 18.5.”

## Discussion

This study evaluated the interplay between BMI and various pain-related outcomes in elderly individuals living with chronic pain. The analysis of PDAS and PCS scores of elderly patients with chronic pain indicated significant reductions in both scores at 3 and 6 months compared to the initial visit. Notably, the low BMI group exhibited significantly higher PCS scores. No significant differences were discerned in PDAS and PCS scores regarding the interaction between BMI category and time point (month).

The PDAS serves as an index for evaluating the ability to perform physical activities of daily living [14]. It constitutes a subjective assessment reflecting four stages, with the content of the interview based on the Cardiovascular Health Study criteria, which are the diagnostic criteria for frailty [21]. The questionnaire content overlaps with that of the CHS criteria for the diagnosis of frailty (including weight loss, muscle strength, fatigue, walking speed, and physical activity). Therefore, PDAS scores in the elderly may reflect not only pain but also frailty. To ensure an accurate evaluation of PDAS, it is crucial to consider the impact of frailty, potentially confounding its interpretation as a pain-specific measure. However, a significant limitation of this study is that we could not quantitatively evaluate frailty, making it challenging to isolate pain-related impairment from broader physical decline. A prior study involving more than 7,000 elderly individuals in Japan highlighted a U-shaped correlation between BMI and frailty [22]. In addition, individual differences in body composition may have influenced the results; individuals with similar BMI may present varying PDAS scores contingent upon their muscle mass and fat mass, particularly resulting in greater score variability within the high BMI group. Age was significantly associated with higher PDAS scores, which may indirectly indicate a relation between older age and frailty.

In the present study, PDAS scores were lower in men. Women are more likely to have functional impairment due to muscle weakness in old age [23]. Previous studies on spinal disease have identified male sex as a predictor of lower PDAS scores [24], which aligns with the findings of the present study. The results also indicated a significant association between declining MMSE scores and lower PDAS scores. Certain PDAS inquiries, such as “visiting friends,” “riding the bus or train,” “engaging in hobby activities,” and “cooking,” are difficult to answer unless cognitive function is preserved to some degree.

To the best of our knowledge, no reports have, to date, analyzed the relationship between PCS and weight loss or appetite loss. A prior study examining BMI and personality tendencies [25] reported that the underweight group exhibited heightened neurotic tendencies and more pronounced anxiety, anger, and depressive tendencies. This suggests that neurotic personality tendencies may underlie the association between low body weight and catastrophic thinking observed in this study, warranting further investigation. PCS scores have been found to be positively correlated with the geriatric depression scale (GDS) scores, which assesses depression in old age [26]. Depression is one of the major causes of anorexia in the elderly [27]. Elevated corticotropin-releasing factor (CRF) in depression leads to anorexia [28], and elderly patients with depression demonstrate dysregulation of the hypothalamic–pituitary–adrenal axis compared to younger patients [29]. Conversely, chronic undernutrition has been associated with an increased risk of depression [30]. This implies a close relationship between catastrophic thinking, loss of appetite, eating disorders, weight loss, and depressive symptoms. However, a significant limitation of this study is the lack of direct GDS measurements, making it difficult to objectively assess depressive symptoms in relation to PCS. Future research incorporating validated depression scales is warranted for a more robust assessment of the relationship between depression and PCS scores in elderly individuals.

MMSE was found to be significantly associated with low PCS scores in this study. Existing literature indicates that MMSE scores in patients with chronic pain fall below the cutoff for mild cognitive impairment (MMSE < 27) [31]. Herein, both PDAS and PCS scores were significantly associated with MMSE, corroborating previous findings. Reports suggest a correlation between cognitive decline and decreased pain tolerance [32], while another study conducted at a Japanese elderly care facility found that patients in the low MMSE group tended to overestimate their pain [33]; therefore, the results of this study may be related to similar mechanisms.

This investigation employed non-invasive, low-cost measures, such as height, weight, and questionnaires, to explore physical and psychological trends in pain severity. Specifically, findings revealed that elderly individuals with low BMI demonstrated stronger catastrophic thinking. This underscores the potential importance of maintaining an appropriate weight in older age [13, 34]. Furthermore, it emphasizes the necessity of addressing nutritional status and body composition in multidisciplinary chronic pain treatment approaches. Future research may yield additional insights by assessing the effectiveness of nutritional interventions in mitigating catastrophic thinking.

Several limitations warrant consideration in this study. The first pertains to selection bias; our department operates as a multidisciplinary pain center within a university hospital, functioning as a referral-only facility. Patients treated in this setting may differ significantly from primary care patients and the broader elderly community, as they often seek treatment after inadequate responses to previous medical care. Second, the analysis did not include the content of the treatment analysis; multidisciplinary treatment is personalized and varies greatly among individuals. Third, categorization of chronic pain sites and pain mechanisms was not strictly performed; this study included all chronic pain, excluding only head and neck pain. Moreover, many patients presented with pain in multiple locations or with generalized pain, leading to challenges in strictly classifying patients based on pain types. Fourth, although weight and MMSE were measured at the initial visit, changes over 3 and 6 months were not accounted for, suggesting the potential for further insights by incorporating these items in future long-term follow-ups.

In conclusion, among elderly patients with chronic pain, the low BMI group displayed significantly higher PCS scores, while PDAS scores did not differ across BMI groups. Additionally, no differences emerged in treatment-related changes over time based on BMI group.

## Supplementary Information

Below is the link to the electronic supplementary material.

Supplementary file1 (DOCX 32 KB)

## Data Availability

The datasets used and/or analyzed during the current study are available from the corresponding author upon reasonable request.
