# Supplementary material for: Association between body mass index and pain outcomes in elderly patients with chronic pain: A retrospective cohort study
Source: J Anesth. 2025 Jul 15;40(1):39–47. doi: 10.1007/s00540-025-03546-2 (PMC12860826; doi:10.1007/s00540-025-03546-2)
Supplement: Supplementary file 1 — Supplementary file1 (DOCX 32 KB) [file 540_2025_3546_MOESM1_ESM.docx]

**SUPPLEMENTAL MATERIAL**

Supplemental Table 1

**PDAS: subgroup analysis; pharmacotherapy (+) subgroup**

|  | **Coefficient** | **P value** | **95% confidence interval** | |
| --- | --- | --- | --- | --- |
| **Month** | | | | |
| 3 month | -4.04 | 0.000 | -6.00 | -2.07 |
| 6 month | -3.09 | 0.007 | -5.32 | -0.85 |
| **BMI** | | | | |
| <18.5 | 2.88 | 0.367 | -3.38 | 9.13 |
| ≥25 | 1.23 | 0.604 | -3.40 | 5.86 |
| **Confounding factor** | | | | |
| Age | 0.39 | 0.013 | 0.08 | 0.69 |
| Male sex | -4.25 | 0.027 | -8.01 | -0.49 |
| CCI | -0.06 | 0.900 | -1.00 | 0.88 |
| MMSE | -1.63 | 0.000 | -2.48 | -0.77 |
| **Interaction: “month**×**BMI group”** | | | | |
| “3month”× “BMI<18.5” | 2.69 | 0.289 | -2.28 | 7.65 |
| “3month”× “BMI≥25” | 2.94 | 0.107 | -0.64 | 6.53 |
| “6month”× “BMI<18.5” | 2.83 | 0.373 | -3.40 | 9.06 |
| “6month”× “BMI≥25” | 3.63 | 0.066 | -0.24 | 7.49 |

BMI: Body Mass Index, CCI: Charlson Comorbidity Index, MMSE: Mini Mental State Examination, PDAS: Pain Disability Assessment Scale, PCS: Pain Catastrophizing Scale

Supplemental Table 2

**PDAS: subgroup analysis; exercise remedy (+) subgroup**

|  | **Coefficient** | **P value** | **95% confidence interval** | |
| --- | --- | --- | --- | --- |
| **Month** | | | | |
| 3 month | -2.56 | 0.003 | -4.28 | -0.85 |
| 6 month | -2.24 | 0.026 | -4.21 | -0.27 |
| **BMI** | | | | |
| <18.5 | 3.37 | 0.334 | -3.46 | 10.19 |
| ≥25 | 2.01 | 0.411 | -2.78 | 6.80 |
| **Confounding factor** | | | | |
| Age | 0.29 | 0.077 | -0.03 | 0.62 |
| Male sex | -4.43 | 0.024 | -8.27 | -0.59 |
| CCI | -0.06 | 0.908 | -1.05 | 0.93 |
| MMSE | -0.99 | 0.065 | -2.04 | 0.06 |
| **Interaction: “month×BMI group”** | | | | |
| “3month”× “BMI<18.5” | 0.02 | 0.994 | -4.76 | 4.79 |
| “3month”× “BMI≥25” | 0.59 | 0.732 | -2.76 | 3.93 |
| “6month”× “BMI<18.5” | 1.12 | 0.696 | -4.52 | 6.77 |
| “6month”× “BMI≥25” | 1.44 | 0.426 | -2.11 | 4.99 |

BMI: Body Mass Index, CCI: Charlson Comorbidity Index, MMSE: Mini Mental State Examination, PDAS: Pain Disability Assessment Scale, PCS: Pain Catastrophizing Scale

Supplemental Table 3

**PCS: subgroup analysis; pharmacotherapy (+) subgroup**

|  | **Coefficient** | **P value** | **95% confidence interval** | |
| --- | --- | --- | --- | --- |
| **Month** | | | | |
| 3 month | -4.07 | 0.000 | -6.08 | -2.05 |
| 6 month | -4.47 | 0.000 | -6.75 | -2.18 |
| **BMI** | | | | |
| <18.5 | 4.67 | 0.120 | -1.21 | 10.54 |
| ≥25 | -1.18 | 0.596 | -5.52 | 3.17 |
| **Confounding factor** | | | | |
| Age | -0.03 | 0.813 | -0.32 | 0.25 |
| Male sex | -0.15 | 0.931 | -3.61 | 3.30 |
| CCI | -0.70 | 0.110 | -1.57 | 0.16 |
| MMSE | -0.92 | 0.021 | -1.71 | -0.14 |
| **Interaction: “month**×**BMI group”** | | | | |
| “3month”× “BMI<18.5” | -2.61 | 0.315 | -7.70 | 2.48 |
| “3month”× “BMI≥25” | 1.60 | 0.393 | -2.07 | 5.27 |
| “6month”× “BMI<18.5” | -2.00 | 0.539 | -8.36 | 4.37 |
| “6month”× “BMI≥25” | 2.63 | 0.193 | -1.33 | 6.58 |

BMI: Body Mass Index, CCI: Charlson Comorbidity Index, MMSE: Mini Mental State Examination, PDAS: Pain Disability Assessment Scale, PCS: Pain Catastrophizing Scale

Supplemental Table 4

**PCS: subgroup analysis; exercise remedy (+) subgroup**

|  | **Coefficient** | **P value** | **95% confidence interval** | |
| --- | --- | --- | --- | --- |
| **Month** | | | | |
| 3 month | -3.70 | 0.000 | -5.68 | -1.73 |
| 6 month | -4.01 | 0.000 | -6.27 | -1.75 |
| **BMI** | | | | |
| <18.5 | 6.77 | 0.042 | 0.26 | 13.28 |
| ≥25 | 1.07 | 0.647 | -3.50 | 5.63 |
| **Confounding factor** | | | | |
| Age | 0.01 | 0.966 | -0.29 | 0.31 |
| Male sex | -0.65 | 0.719 | -4.16 | 2.87 |
| CCI | -0.76 | 0.099 | -1.67 | 0.14 |
| MMSE | -0.38 | 0.434 | -1.34 | 0.58 |
| **Interaction: “month**×**BMI group”** | | | | |
| “3month”× “BMI<18.5” | -1.66 | 0.554 | -7.16 | 3.84 |
| “3month”× “BMI≥25” | 0.92 | 0.638 | -2.92 | 4.77 |
| “6month”× “BMI<18.5” | -2.82 | 0.394 | -9.29 | 3.66 |
| “6month”× “BMI≥25” | 2.18 | 0.295 | -1.90 | 6.26 |

BMI: Body Mass Index, CCI: Charlson Comorbidity Index, MMSE: Mini Mental State Examination, PDAS: Pain Disability Assessment Scale, PCS: Pain Catastrophizing Scale

Supplemental Table 5

**Mixed effects model (PCS-ruminate)**

|  | **Coefficient** | **P value** | **95% confidence interval** | |
| --- | --- | --- | --- | --- |
| **Month** | | | | |
| 3 month | -1.45 | <0.001 | -2.21 | -0.69 |
| 6 month | -1.60 | <0.001 | -2.50 | -0.71 |
| **BMI** | | | | |
| <18.5 | 2.13 | 0.059 | -0.08 | 4.34 |
| ≥25 | 0.01 | 0.992 | -1.54 | 1.55 |
| **Confounding factor** | | | | |
| Age | -0.04 | 0.375 | -0.14 | 0.05 |
| Male sex | -0.32 | 0.595 | -1.48 | 0.85 |
| CCI | -0.32 | 0.042 | -0.63 | -0.01 |
| MMSE | -0.25 | 0.057 | -0.52 | 0.01 |
| **Interaction: “month**×**BMI group”** | | | | |
| “3month”× “BMI<18.5” | -0.47 | 0.664 | -2.57 | 1.63 |
| “3month”× “BMI≥25” | 0.83 | 0.262 | -0.62 | 2.28 |
| “6month”× “BMI<18.5” | 0.31 | 0.812 | -2.27 | 2.89 |
| “6month”× “BMI≥25” | 0.28 | 0.733 | -1.32 | 1.87 |

BMI: Body Mass Index, CCI: Charlson Comorbidity Index, MMSE: Mini Mental State Examination, PDAS: Pain Disability Assessment Scale, PCS: Pain Catastrophizing Scale

Supplemental Table 6

**Mixed effects model (PCS-helplessness)**

|  | **Coefficient** | **P value** | **95% confidence interval** | |
| --- | --- | --- | --- | --- |
| **Month** | | | | |
| 3 month | -1.79 | <0.001 | -2.56 | -1.01 |
| 6 month | -1.32 | 0.005 | -2.23 | -0.40 |
| **BMI** | | | | |
| <18.5 | 2.87 | 0.022 | 0.41 | 5.33 |
| ≥25 | 0.15 | 0.866 | -1.57 | 1.87 |
| **Confounding factor** | | | | |
| Age | 0.02 | 0.751 | -0.09 | 0.13 |
| Male sex | -0.13 | 0.851 | -1.46 | 1.21 |
| CCI | -0.07 | 0.714 | -0.42 | 0.29 |
| MMSE | -0.52 | 0.001 | -0.82 | -0.22 |
| **Interaction: “month×BMI group”** | | | | |
| “3month”× “BMI<18.5” | -0.64 | 0.556 | -2.77 | 1.49 |
| “3month”× “BMI≥25” | 0.59 | 0.431 | -0.88 | 2.07 |
| “6month”× “BMI<18.5” | -1.46 | 0.277 | -4.09 | 1.17 |
| “6month”× “BMI≥25” | 0.85 | 0.306 | -0.78 | 2.47 |

BMI: Body Mass Index, CCI: Charlson Comorbidity Index, MMSE: Mini Mental State Examination, PDAS: Pain Disability Assessment Scale, PCS: Pain Catastrophizing Scale

Supplemental Table 7

**Mixed effects model (PCS- catastrophizing)**

|  | **Coefficient** | **P value** | **95% confidence interval** | |
| --- | --- | --- | --- | --- |
| **Month** | | | | |
| 3 month | -0.57 | 0.059 | -1.15 | 0.02 |
| 6 month | -0.78 | 0.027 | -1.47 | -0.09 |
| **BMI** | | | | |
| <18.5 | 1.62 | 0.058 | -0.06 | 3.30 |
| ≥25 | 0.23 | 0.698 | -0.94 | 1.41 |
| **Confounding factor** | | | | |
| Age | 0.01 | 0.737 | -0.06 | 0.09 |
| Male sex | 0.08 | 0.866 | -0.81 | 0.96 |
| CCI | -0.02 | 0.847 | -0.26 | 0.21 |
| MMSE | -0.14 | 0.151 | -0.34 | 0.05 |
| **Interaction: “month**×**BMI group”** | | | | |
| “3month”× “BMI<18.5” | -1.97 | 0.018 | -3.59 | -0.34 |
| “3month”× “BMI≥25” | 0.09 | 0.873 | -1.03 | 1.21 |
| “6month”× “BMI<18.5” | -1.20 | 0.240 | -3.19 | 0.80 |
| “6month”× “BMI≥25” | 0.92 | 0.144 | -0.31 | 2.15 |

BMI: Body Mass Index, CCI: Charlson Comorbidity Index, MMSE: Mini Mental State Examination, PDAS: Pain Disability Assessment Scale, PCS: Pain Catastrophizing Scale
